# Sérgio Ferreira beyond Pharmacology: His Role as a Science Communicator

**DOI:** 10.3390/toxins15090516

**Published:** 2023-08-24

**Authors:** Cristiane Flora Villarreal, Paulo Gustavo Barboni Dantas Nascimento, Beatriz Rossetti Ferreira, Mani Indiana Funez

**Affiliations:** 1 School of Pharmacy, Federal University of Bahia, Salvador 40170-115, BA, Brazil; cfv@ufba.br; 2 School of Ceilândia, University of Brasília, Brasília 72220-275, FD, Brazil; pbarboni@unb.br; 3 Ribeirao Preto College of Nursing, University of Sao Paulo, Ribeirao Preto 14040-902, SP, Brazil; brferrei@usp.br

Historically, toxins from animal venoms have contributed significantly to the discovery of new drugs, as illustrated by captopril, the first drug developed from an animal toxin approved for human use [1]. Captopril was synthesized from a peptide present in snake venom and was a milestone in the development of angiotensin-converting enzyme inhibitors, now considered a pillar class of antihypertensive drugs used in the modern pharmacotherapy of arterial hypertension and heart failure [2]. The discovery of captopril originated in the pioneering studies of Professor Sérgio Henrique Ferreira, physician and researcher at the Ribeirão Preto School of Medicine, belonging to the University of São Paulo (FMRP-USP, as per its Portuguese acronym), and one of the most relevant pharmacologists in Brazil. In the 1960s, while still a graduate student at the Pharmacology Department of FMRP under the supervision of Professor Mauricio Rocha e Silva, Professor Sérgio identified and characterized a component of the *Bothrops jararaca* venom capable of inhibiting the degradation of bradykinin. He named this component “bradykinin potentiating factor”, which was, in the following years, proven to be an angiotensin-converting enzyme inhibitor and gave origin to captopril [3]. Accordingly, the work of Professor Sérgio Ferreira represents a milestone in the bioprospecting of venoms and toxins for drug development.

In fact, the academic career of Professor Sérgio has left a vast legacy for scientific knowledge worldwide, as illustrated by his contributions to discoveries regarding the mechanism of action of non-steroidal anti-inflammatory drugs, the mechanism of action of dipyrone, peripheral analgesia of opioids, peripheral mechanisms of pain information processing and pain chronification, among other topics of global relevance [4,5,6,7,8,9,10,11,12,13,14,15,16]. His brilliant performance as a pharmacologist and his contributions to science have been widely recognized by the world scientific community, as illustrated by the awards he has received (24 awards and titles), and his more than 300 scientific articles, 3 books and 51 book chapters.

Nevertheless, it is important to emphasize that his academic contributions go beyond the discoveries and advances in Pharmacology, as Professor Sérgio is one of Brazil’s pioneers in actions focused on disseminating science. For Professor Sérgio, doing science is not enough; it is also necessary to spread the knowledge generated to the general population. He believed that producing scientific knowledge is as important as communicating it to society, and this is a fundamental point for the strengthening of science. In addition, science communication, which includes helping people to understand the knowledge generated from research, is part of the commitment of researchers to society. In the year 2000, this vision led Professor Sérgio to launch a project of science diffusion through the Internet called DOL–Boletim Dor Online. The project was established and has developed in the academic environment as a society-oriented action, translating scientific knowledge into accessible language. It is conducted by professors as well as undergraduate and graduate students. The goal of DOL is to disseminate, in Portuguese, current pertinent, relevant and general interest content about pain and pain control studies, based on findings published in scientific journals of worldwide relevance.

The DOL–Dor Online website was a precursor in the diffusion of quality scientific knowledge to the population in Brazil. In 2000, when the project was launched, the internet in the country was in its infancy and could not be used to its full potential. At that time, the concept and understanding of the relevance of science dissemination and education was incipient in the sphere of public health policies and even for the scientific community in the country. Therefore, Professor Sérgio Ferreira was at the forefront of the scientific dissemination movements in Brazil.

In addition, at the time of its implementation, DOL was fully aligned and synchronized with the recommendations of the International Association for the Study of Pain on the need to expand pain education programs, especially in developing countries. Epidemiological studies from the late 1990s and early 2000s revealed that pain education and clinical management in developing countries lagged behind richer areas of the world [17]. This reality drove the establishment of the IASP Task Force for Developing Countries in 2002, to improve pain education and control in these countries. Accordingly, the actions developed in the DOL project are aligned, since its inception, with the global demands for strategies aimed at improving pain management in society.

The first DOL newsletter was released in August 2000. The idea was to create a virtual monthly newsletter, which would be sent by e-mail to interested parties in the form of a bulletin, in addition to being published on the website (http://www.dol.inf.br, accessed on 15 June 2023). Currently, the bulletin editions continue to be published monthly on the website, but also in the electronic journal “Dor On Line”, available at the Periodical Portal of the University of Brasília, in epub format (https://periodicos.unb.br/index.php/dol, accessed on 15 June 2023). Moreover, in recent years, to keep up with social changes in terms of digital behavior, the dissemination of DOL has been expanded to other digital media in addition to the portal, such as Facebook (https://www.facebook.com/DolDorOnLine/, accessed on 15 June 2023) and Instagram (@dol.doronline).

DOL has been on the air for 23 years, monthly and uninterruptedly, enabling the communication of original research results to the scientific community, health professionals, students, patients and their families and other interested parties. It constitutes an important scientific dissemination tool for Portuguese-speaking countries, fostering discussions about pain studies and their daily and scientific implications. The DOL portal has constant access, with about 3000 visitors per month. The origin of these accesses is scattered, with a large part coming from Brazil, but also from other countries, such as the United States, Germany, England and France, among others, thus reaching readers from various parts of the world.

The journal maintains its publications based on varied and highly credible sources in the scientific environment, thus ensuring the plurality and quality of the content published. Among the sources of information used to compose the DOL editions, one can mention several journals with high impact factors (IF), such as *JAMA–The Journal of The American Medical Association* (IF 157), *Lancet* (IF 202.7), *The New England Journal of Medicine* (IF 176), *Nature* (IF 69.5), *Science* (IF 63.7), *Pain* (IF 7.9), among others. The themes addressed in the issues of DOL cover topics of general interest in this field, such as “Types of Pain”, “Pain Treatments”, “New Targets and Drugs” and “Pathophysiological Mechanisms”. In addition, the Bulletin encourages discussion of contemporary issues of society, establishing its intersection in the field of pain, as exemplified by publications on “COVID-19 and Pain”, “Telemedicine and Telehealth in Pain”, “Racial Disparities in Pain Management”, and “Pain in the Transgender Individual”. This approach allows the project to remain updated and relevant, aligned with current discussions in society, and to be continuously integrated with new global demands.

In this context, it is important to underline that, despite the enormous scientific advances observed in recent decades, the task of improving pain control is still a relevant global demand, especially in less developed countries. Among the barriers to adequate pain control in developing countries, one can find first, the lack of education of health professionals on the subject (91%); and, second, the lack of public policies (74%), followed by fear of opioid dependence, high cost of drugs and low patient compliance, among others [18]. Given this scenario, the DOL project was inserted into undergraduate and graduate teaching, aiming at strengthening the training of health professionals on the topic of pain. The project acts in the training of students in terms of pain and scientific journalism, thus favoring the development of critical awareness, obtained from the discussions of scientific papers and collaborative production of the monthly editions. About 130 undergraduate students are enrolled in the activities of the project every year. These students come from multiple subareas of the health sciences, including nursing, pharmacy, physiotherapy, speech therapy, occupational therapy, collective health, dentistry and medicine. Additionally, about 50 graduate students at the master’s and doctorate levels also contribute every year. The educational impact of the DOL project is twofold: the communication vehicle is assertive in delivering its content to the audience, while its elaboration is an effective educational tool for Health students who contribute with such content.

Besides assisting the training of health professionals, who will become more capable of dealing with pain management, the insertion of this project in undergraduate and graduate teaching has been contributing to the acquisition of skills related to Education for Communication [19]. The DOL project promotes health education using different technology tools, which agrees with the principles of educommunication. Educommunication is a movement that emerged in Latin America in the late 1960s that advocates that education and communication are intertwined and must be linked to the cultural and political dimensions of society. Tárcia et al. recently showed positive impacts of using the transmedia educommunication method in elementary schools from Portuguese-speaking countries [20]. These authors suggest that transmedia educommunication is relevant to student-centered teaching, in an approach to education in which students are active participants in the learning process, and not passive recipients of information. The positive impact of educommunication tools on Brazilian public health has also been verified, as shown by Caitano et al. [21] using Massive Open Online Courses (MOOCs) in a Virtual Learning Environment in response to the syphilis epidemic in Brazil. According to Goldberg and Crocombe [22], MOOCS can increase the health literacy of the general public. They can also provide interprofessional education while employing innovative teaching models focused on patient- and family-centered care. As described by Nascimento et al. [19], the workflow resulting in each DOL edition can be performed in a Virtual Learning Environment, employing several educommunication tools, mimicking some aspects found in MOOCS, though on a much smaller scale. Health students participating in the DOL project are also subject to an innovative teaching tool in health education, which will strengthen their knowledge on topics such as prevention and treatment of chronic pain, new drugs and analgesic techniques, analgesic toxins, among other topics relevant to the Study of Pain and Pain Education. The project is particularly important when considering the worldwide gap in pain education, and its impacts on pain management, which are even more relevant in developing countries such as Brazil and other Portuguese-speaking countries.

The DOL project is one of the actions idealized and carried out by Professor Sérgio Ferreira for disseminating scientific knowledge to society, as well as for strengthening the training of human resources in Brazil. His trajectory illustrates that, far beyond his contributions to science, Professor Sérgio left as a legacy his example as a scientist. His vision of the scientist as a professional unified and committed to society, and not apart from it, is a continuous source of inspiration and motivation for the generations of researchers who succeeded him, and for the new generations yet to come.
